# Assessment of genotoxic potential of the insecticide Dichlorvos using cytogenetic assay

**DOI:** 10.2478/intox-2013-0014

**Published:** 2013-06

**Authors:** Nazia Nazam, Mohammad Iqbal Lone, Sibhghatulla Shaikh, Waseem Ahmad

**Affiliations:** Gene-Tox Laboratory, Division of Genetics, Department of Zoology, Aligarh Muslim University, Aligarh, 202002, UP, India

**Keywords:** genotoxicity, micronucleus, chromosome aberration, *Mus musculus*

## Abstract

The possible genotoxic activity of Dichlorvos (2,2-Dichlorovinyl-O,O-dimethyl phosphate/DDVP, CAS No. 62-73-7), an organophosphorus insecticide was investigated employing three cytogenetic end points, i.e. micronucleus (MN) assay, mitotic indices (MI) and chromosome abberation (CA) analysis *in vivo*. The assays were carried out in hematopoietic bone marrow cells of *Mus musculus* at concentrations of 10, 20 and 30% of LD_50_ for intraperitoneal (ip) administration, corresponding to 0.06, 0.08 and 0.13 mg/kg Bwt, respectively. The normal control group received single ip dose of distilled water (2 ml/100 g Bwt), while animals of the positive group were injected with cyclophosphamide, a model mutagen (40 mg/kg Bwt) under identical conditions. The animals were sacrificed 24, 48 and 72 hrs post treatment. Under the present experimental conditions, there was no evidence of significant increase of MN frequencies at any dose or sampling time in polychromatic (PCE) and normochromatic (NCE) erythrocytes. The PCE/NCE ratio was not notably affected; however, a slight depression in prolonged exposure (48, 72 hr) intervals and a slight increase at the 24 hr interval were observed. Cells with various structural chromosome aberrations were noted but no significant (*p<*0.05; Man-Whitney U-test) differences in the frequencies of CA or mitotic indices (*p<*0.05; χ^2^ test) were observed between Dichlorvos treated groups and the normal control group at doses or time intervals used. The results of the present investigation reflects a negative in vivo genotoxic potential of Dichlorvos at sublethal doses in bone marrow cells. Further studies are underway to confirm the presence or absence of genotoxic activity since compounds negative in genotoxic evaluation are susceptible of being carcinogens triggering cancer by genotoxic or non–genotoxic mechanisms.

## Introduction

The concern over potential hazards of organophosphorus (OP) pesticides was raised as soon as trimethyl phosphate was reported to be mutagenic in mice (Epstein *et al.*, 1970). Since then these became most favored and contributed be so till recently (Tripathi & Srivastav, 2010). The worldwide increase of OP compunds in food and fiber production and their extended use in the control of major disease carrying vectors make them highly alarming (Rahman *et al.*, 2002; Chen *et al.*, 1999). Their effect on non target organisms including humans further necessitated their assessment (Karabay & Oguz, 2005; Chaudhuri *et al.*, 1999). This class of compounds is implicated in environmental pollution, health hazards and human poisoning (Bradberry et. al., 2005), and more seriously, they possess biological activity that may influence proliferating cells and cause disturbance of the genetic material. Initial studies (Blasiak *et al.*, 1999) pointed to their possible genotoxicity since many of these compounds are known to be mutagenic. Some of the OP compounds have been in use for a long time yet without significant attempts of assessment; one such compound is Dichlorvos.

Dichlorvos (2,2-Dichlorovinyl-O,O-dimethyl phosphate) is a synthetic OP insecticide which can cause exposure via air, water or food and can readily be absorbed through all routes of exposures (Raheja and Gill, 2002). The International Agency for Research on Cancer (IARC) has classified Dichlorvos as possible carcinogen to humans – Group 2B (Ishmael *et al.*, 2006; IARC, 1991). The Environmental Protection Agency (EPA) has also classified Dichlorvos with a toxicity of class I, meaning a highly toxic chemical with a potential to cause cancer and tumors in diverse mammals (EPA, 1991a; b). These reports are serious enough to warrant a thorough genotoxic evaluation of the most widely used Dichlorvos.

To assess the potential genotoxicity of a compound, its ability to cause DNA damage can effectively be evaluated employing various cytogenetic end points (Repetto *et al.*, 2001), especially in small mammals (Topashka-Ancheva *et al.*, 2003). We employed two eukaryotic mutagenicity assays, namely micronucleus (MN) test and the chromosome aberration (CA) assay in bone marrow cells of *Mus musculus* for assessing the genotoxic and mutagenic potential of Dichlorvos. These are standard bioassays that best reflect the delicate balance between pathways for activation and inactivation of chemicals in mammals, including human beings (Bakare *et al.*, 2009).

## Materials and methods

The protocols related to the parameters used were in accordance with the international guidelines for *in vivo* genotoxicity testing in mammalian models (EPA, 2005; OECD, 1997). The experimental animals were procured and sacrificed according to the University Ethical Regulations and standard chemicals were used throughout the experiments.

### Test chemicals

The test compound Dichlorvos (CAS No. 62-73-7; 2,2-Dichlorovinyl-O,O-dimethyl phosphate, 76% EC DDVP insecticide, Crystal phosphate Limited, India) with a purity of 98% was used for preparing stock solution. Cyclophosphamide (CAS No. 6055-19-2) and Colchicine (CAS No. 64-86-8) were purchased from HIMEDIA, India. All other chemicals and reagents used were of analytical grade.

### Animals and husbandry

Swiss albino male mice, *Mus musculus* averaging 30 g (8–10 weak old), were used in the study. The animals were kept in environmentally controlled conditions at a temperature of 22±1 °C and relative humidity of 30–70%, on a 12/12 h light/dark cycle. Commercially available sterilized pellet (Amrut Laboratory animal feed) and quality drinking water were offered *ad libitum*. After 5 days of acclimatization, random allocation of animals to the exposed groups was carried out.

### Study design and distribution of animals

Male mice (75 in number) were allocated to 5 groups, each containing 5 animals labeled I–V. Animals of groups III–V were treated intraperitoneally once with an individual dose of 0.06, 0.08 and 0.13 mg/kg Bwt of DDVP, respectively, dissolved in distilled water. Groups I and II were respectively treated with the solvent/vehicle, only i.e. distilled water (2 ml/100 g Bwt) to be used as normal control and with cyclophosphamide (40 mg/kg Bwt) to serve as positive control. The dose regimen was maintained for multiple intervals: 24, 48 and 72 h before sacrifice. All observations were replicated thrice for varied observations.

### MN evaluation

The procedural details followed now famous Schmid (1975) technique. The animals were sacrificed by cervical dislocation after completion of exposure. Both femurs were removed carefully and the marrow flushed with Foetal Calf Serum (Sigma Aldrich, Germany). The cells were subsequently centrifuged at 1000 rpm for 5 min and the sediment suspended in 50 µl fresh FCS, and used as smear on grease-free slides. Methanol-fixed slides were stained with May-Grunwald and Giemsa stains. Independently coded slides were tested for analyzable MN. The final observation and photography was carried out at 100× (Olympus U – PMTV microscope mounted with optical zoom camera) using oil immersion. At least 2000 immature erythrocytes per animal were scored to assess the incidence of MN induction. The differential staining of PCEs – polychromatic erythrocytes (bluish-purple) and normochromatic erythrocytes (NCEs, pinkish-orange) assisted the differentiation between the two types of erythrocytes for relevant comparison.

### Chromosome aberration assay

The metaphase chromosomes were prepared using the Preston *et al.* (1987) method. An aqueous solution of colchicine (4 mg/kg Bwt), 2 h prior to sacrificing the animal by cervical dislocation, was injected intraperitoneally. The marrow cells were aspirated in pre-warm KCL (0.075 M) solution, homogenized and the suspension was incubated at 37 °C for 20 min followed by centrifugation at 1000 rpm for 10 min. The pellet was fixed in cold Cornoy's fixative (methanol: glacial acetic acid, 3:1 V/V) and dropped onto clean pre-chilled glass slide in 30% ethanol and air-dried. The staining achieved in 5% buffered Giemsa (pH 7.0). Only properly separated metaphases were analyzed for chromosomal aberrations (CA) blindly and finally photographed at 100X (Olympus U – PMTV microscope mounted with optical zoom camera), under oil immersion. At least 100 well spread metaphase cells/mouse were observed. The metaphase cells from approximately 1000 cells per concentration per animal both in exposed and control replicates, expressed in percentage were considered for mitotic indices (MI).

### Statistical analysis

The data obtained were expressed as percentage frequency and mean ± standard error. The SPSS^®^16.0 (Statistical Package for Social Science) and Med Calc 12.0 softwares were used for statistical analysis. Significance at the different dose levels and time periods in MNT and CA assays was tested by Man-Whitney-U (MW-U) test. Data on MI were expressed with 95% confidence limits and χ^2^ comparison of proportion was used for testing the significance. Difference between the control and individual exposed groups were analyzed at the 0.05 probability level.

## Results

**Figure 1:**
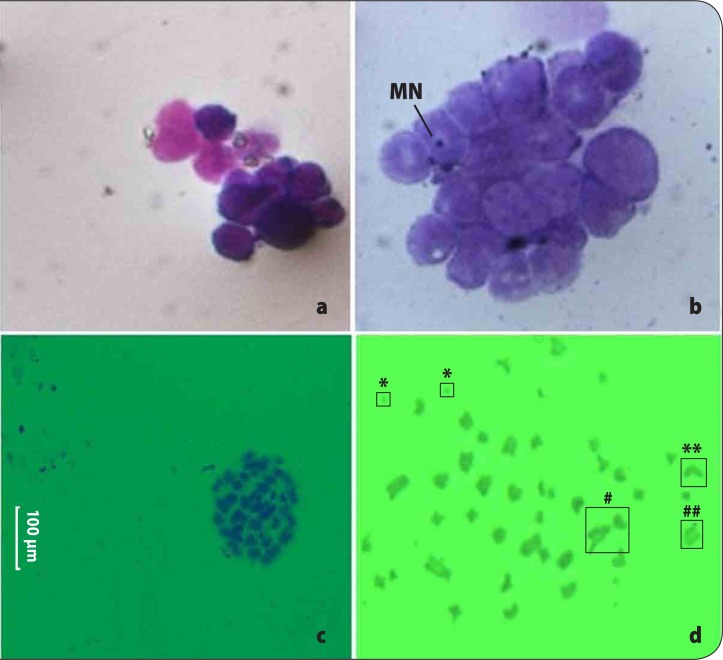
Mice bone marrow cells showing (a) normal polychromatic erythrocytes – PCE (Blue), normal normochromatic erythrocytes – NCE (Pink); (b) micronucleated – MNPCE (marked); (c) normal metaphase chromosomes; (d) metaphase plate showing chromosomal aberrations – fragment (*), deletion (**), chromosome break (#), gap (##) post exposure. Magnification 100×

The MN and CA were carried out for each test group per sacrifice interval and analysis and are given in Table 1 and Table 2 along with the representative figures of each assay in Figure 1, while Table 3 presents the MI of scored metaphase cells. The mean value for five male animals per concentration is represented by each data point in Table 1 for MN assay. The MN study demonstrates that the number of polychromatic erythrocytes containing MN (MNPCEs) at each dose of DDVP and time interval was not significantly increased above the concurrently run normal control frequencies (*p<*0.05; MW-U). The MNPCEs frequency in the positive control group registered a significant increase at all time periods compared to normal control, demonstrating the expected activity and sensitivity of the experimental system. The indicator of cytotoxicity, the PCE/NCE (P/N), was not affected in Dichlorvos treated animals; however the slight depression at 48 and 72 hrs and the increase at the 24 hr interval reflect normal variability rather than bone marrow toxicity. The corresponding P/N observation was found to be significantly decreased in the CPA treated positive group at 24-hr, 48-hr and 72-hr treatment periods.

**Table 1:** The micronucleus assay of mice bone marrow exposed to Dichlorvos (Mean MNPCE % ± SE at three different time intervals).

	Time (hr)	MNPCE(% ± SE)	MNWCE(% ± SE )	PCE/NCE
**Control groups**				
**Normal** (Distilled water)	24	0.49±0.05	0.19±0.09	0.743
	48	0.46±0.06	0.23±0.05	0.754
	72	0.48±0.06	0.19±0.09	0.748
**Positive - CPA** (40mg /kg Bwt)	24	4.69±1.38*	1.00±0.39	0.539*
	48	5.62±1.55*	1.58±0.87	0.508*
	72	4.27±1.30*	0.87±0.21	0.527*
**Exposed groups - Dichlorvos (mg/kg Bwt)**				
0.06	24	0.55±0.03	0.17±0.09	0.745
	48	0.43±0.07	0.17±0.09	0.750
	72	0.48±0.06	0.14±0.08	0.730
0.08	24	0.51 ±0.05	0.14±0.08	0.746
	48	0.45±0.07	0.21 ±0.09	0.752
	72	0.46±0.06	0.11 ±0.07	0.737
0.13	24	0.49±0.05	0.19±0.09	0.745
	48	0.45±0.07	0.19±0.09	0.721
	72	0.46±0.06	0.11 ±0.07	0.730

* The values are significant at 0.05 (MW-U test)

**Table 2:** Effect of various doses of Dichlorvos on the metaphase chromosomes of bone marrow cells of *Mus musculus* using multiple doses and durations.

			Number and type of chromosomal aberrations														
	Time (hr)	SMC	Breaks		Rings		Exchanges		Dicentrics		S & P		Gaps		Total (excluding gap)		Total (%±SE)
**Control groups**			**no.**	**%**	**no.**	**%**	**no.**	**%**	**no.**	**%**	**no.**	**%**	**no.**	**%**	**no.**	**%**	
**Normal**(Distilled Water)	24	497	2	0.4	1	0.2	0	0	1	0.2	0	0	0	0	4	0.8	1.06±0.53
	48	492	1	0.2	0	0	0	0	1	0.2	0	0	1	0.2	2	0.4	0.73±0.07
	72	495	0	0	1	0.2	0	0	0	0	0	0	0	0	1	0.2	0.47±0.06
**Positive - CPA**(40 mg/kg Bwt)	24	488	18	3.68	9	1.84	1	0.2	3	0.61	8	1.63	1	0.2	39	7.99	12.96±3.01*
	48	490	29	5.91	13	2.65	3	0.61	1	0.2	9	1.83	0	0	55	11.22	18.35±4.08*
	72	493	19	3.85	22	4.46	6	1.22	0	0	6	1.21	1	0.2	53	10.75	17.56±3.93*
**Exposed groups - Dichlorvos (mg/kg Bwt)**																	
**0.06**	24	487	3	0.61	1	0.2	1	0.2	0	0	0	0	1	0.2	5	1.02	1.11±0.92
	48	484	1	0.2	0	0	0	0	0	0	1	0.2	0	0	2	0.41	0.73±0.08
	72	468	1	0.21	0	0	0	0	0	0	0	0.21	0	0	1	0.21	0.28±0.06
**0.08**	24	490	2	0.4	1	0.2	0	0	0	0	0	0	0	0	3	0.61	0.93±0.28
	48	479	2	0.41	0	0	0	0	0	0	0	0	0	0	2	0.41	0.73±0.08
	72	484	1	0.2	0	0	1	0.2	0	0	0	0	0	0	2	0.41	0.48±0.06
**0.13**	24	480	0	0	1	0.2	0	0	1	0.2	1	0.2	0	0	3	0.625	0.95±0.31
	48	491	0	0	1	0.2	1	0.2	0	0	0	0	1	0.2	2	0.407	0.74±0.08
	72	482	0	0	0	0	0	0	0	0	1	0.2	0	0	1	0.207	0.48±0.06

* Values are significant at 0.05 (MW-U test); SMC= Scored Metaphase Cells; S = Stickiness; P = Pulverization.

**Table 3:** Mitotic index profiles in bone marrow cells of *Mus musculus* exposed to Dichlorvos.

	Time	Totalcell No.	No. of dividing cells	MI (%)
**Control group**				
**Normal** (Distilled water)	24	5000	244	4.88
	48	5000	248	4.96
	72	5000	251	5.02
**Positive - CPA** (40mg/kgBwt)	24	5000	126	2.52*
	48	5000	143	2.86*
	72	5000	158	3.16*
**Exposed group - Dichlorvos (mg/kg Bwt)**				
**0.06**	24	5000	245	4.90
	48	5000	249	4.98
	72	5000	261	5.22
**0.08**	24	5000	242	4.84
	48	5000	251	5.02
	72	5000	252	5.04
**0.13**	24	5000	238	4.76
	48	5000	253	5.06
	72	5000	246	4.92

* Values are significant at 0.05 (χ^2^ test)

Many types of aberrations were observed in DDVP and CPA treated replicates. That included breaks, fragments, exchanges and multiple aberrations like dicentrics, gaps, stickiness and pulverization. Data of structural chromosomal analysis in Table 2 show no significant differences (*p<*0.05) in the frequencies of chromosome aberrations between Dichlorvos treated and normal control group at either dose level or time interval used.

The present investigation also showed a lack of significant difference in the percentage of MI in bone marrow cells at any dose or duration between the stressed animals and the normal controls as summarized in Table 3. Expectedly, the MI significantly decreased in the positively treated group. A difference of 4.05% (*p=*0.0423) was obtained when the positive control contrasted with the normal, showing the presence of a significant difference between them and thus a decrease in MI (%) in the positive group.

## Discussion

The effect of spindle poison and clastogenic chemical could be detected in bone marrow within 24–48 hr post treatment (Vanparys *et al.*, 1992), so the time assigned in this study allowed for a sufficient window period for detecting the clastogen and aneugen. But the results from the MN and CA assessment showed that Dichlorvos failed to elicit any significant MN induction or chromosome anomalies at the dose or duration of treatment. The study is noted for the lack of significant difference in the MI profiles of the DDVP group and the normal control. Consequently, no clastogenic or aneugenic effect is expected in the treatment regimen in bone marrow cells. These findings reciprocate earlier investigations reporting a lack of chromosome damage (Schop *et al.*, 1990; Ramel *et al.*, 1980).

Cytotoxic effects are measured by P/N ratio, indicating alteration in erythropoiesis. This parameter was also tested in our studies. The concentrations of DDVP did not significantly reduce the P/N ratio. On close examination, the slight depression at longer durations and the increase at 24 hr interval reflect normal variability rather than bone marrow toxicity. In contrast, a prominent and significant decrease in P/N ratio was noted in the CPA treated positive control, which is an established antitumor agent. CPA is a known genotoxicant in bone marrow of mice and rats (Gollapudi *et al.*, 1984). Witt *et al.* (2008) suggested the decrease to be due to cavity formation in bone marrow when there are cytotoxic effects in cell division, or it may be caused by maturation of nucleated cells.

Some authors prefer the simultaneous cytotoxicity determination by CA assay along with the mitotic index (MI). The enhanced MI indicates interference with the spindle apparatus or with protein synthesis. This can increase the cell proliferative activity or decrease the MI, indicating a lower number of cells completing the cell cycle. A high proportion of cells belonging to the resting stage of the cell cycle are observed as a result (Verma & Purnima, 1992). The lack of significant decrease or increase in MI of the exposed group contrasting with the control is suggestive that DDVP neither inhibits nor induces mitotic progression. The negative results obtained in our study reinstate the earlier findings that Dichlorvos can be accepted as a non-toxic agent under conditions relevant to human exposure.

Ashby (1983) divulged various factors that can modulate the *in vivo* expression of DDVP. Its rapid conversion by esterase hydrolysis presumably contributes to the negative response in short-term studies (NTP, 1989). Due to the common occurrence of esterase enzymes in mammalian cells and in blood, the hydrolytic pathway predominates over demethylation, which is usually responsible for the genotoxic activity of dichlorvos *in vitro* (Bremmer *et al.*, 1988). Dichlorvos administered rodents reflecting the carcinogenic potential as neoplastic responses on the hematopoietic system and various tissues were also reported (Chan *et al.*, 1991).

The present and past observations thus assessed Dichlorvos as a suspected carcinogen, since compounds negative in genotoxicity assessments may be carcinogen or non-carcinogens capable of triggering cancer by genotoxic or non–genotoxic mechanisms. More importantly, the carcinogen with a non-genotoxic mechanism may score negative and so be the false negative in genotoxicity tests. A recent database by Kirkland *et al.* (2005) further emphasizes the mechanism of action of carcinogenicity of 80% of false negative substances to be non-genotoxic. Additional genotoxicity testing is recommended since Dichlorvos is negative in the standard genotoxicity test battery with insufficient evidence to establish a non genotoxic mechanism.
